# Naïve conceptions about multimedia learning: a study on primary school textbooks

**DOI:** 10.3389/fpsyg.2013.00450

**Published:** 2013-07-30

**Authors:** Barbara Colombo, Alessandro Antonietti

**Affiliations:** Psychology Department, Catholic University of the Sacred HeartMilano, Italy

**Keywords:** multimedia learning, naïve conceptions, metacognition, illustrated textbooks, primary school, interview study, teachers, illustrators

## Abstract

**HIGHLIGHTS**

- This interview study explores beliefs about the instructional role of illustrations
- We compared illustrators', teachers', students' and common people's ideas
- Participants' responses were internally coherent and close to multimedia learning theory
- We propose and discuss an integrated multimedia learning model

An interview study, based on specific pictures taken from textbooks used in primary schools, was carried out to investigate illustrators', teachers', students', and common people's beliefs about the role that illustrations play in facilitating learning. Participants' responses were internally coherent, indicating a systematic nature of the underlying naïve conceptions. Findings disprove Mayer's pessimistic claim that laypersons' conceptions of multimedia learning fail to match experimentally supported principles and theories. On the contrary, interviewees spontaneously came very close to the multimedia learning theory, which states that students learn better from pictures, which fit specific cognitive principles. Implications for school instruction are highlighted.

## Introduction

The purpose of the present study is to investigate people's naïve conceptions about the mental processes supporting the efficacy of multimedia artifacts to be employed in instructional settings. To explain the rationale underlying the research project, the concept of multimedia learning will first be described. Then, the issue of the folk conceptions about instructional devices will be addressed. The third part of the Introduction is aimed at linking such conceptions to multimedia learning. Finally, we will present the specific perspective concerning naïve conceptions about multimedia learning on which our study is based.

### Multimedia learning

The cognitive theory of multimedia learning, proposed by Mayer (2001, 2005), can be assumed as a basis to support the benefits produced by multimedia in school instruction. Such a theory has its root in the Multiple Representation Principle (Mayer and Anderson, 1992), which states that it is better to present an explanation in words and pictures than solely in words, since two modes of representation allow learners to extract information from two different sources (so that, if learners are inefficient in managing one of them, they can rely on the other) and to store it in two distinct systems (so that, if the verbal track is disrupted, the visual one can keep information in memory and vice versa).

Mayer identified seven specific principles aimed at explaining how and why multimedia learning is actually effective. The *spatial contiguity* principle states that students learn better when corresponding words and pictures are presented close to, rather than far from, each other on the page or on the screen. Similarly, the *temporal contiguity* principle states that learning is enhanced when corresponding words and pictures are presented simultaneously rather than successively. The *coherence* principle claims that students learn better when extraneous visual material is excluded rather than included. The *modality* principle states that students learn better from animation and narration than from animation and on-screen text. The *redundancy* principle asserts that students learn better from animation and narration than from animation, narration, and text. Mayer further hypothesized an *individual difference* principle, according to which multimedia effects are stronger for low-knowledge than for high-knowledge learners and for high-spatial rather than for low-spatial learners.

Schnotz (2005) proposed new principles to integrate those proposed by Mayer: the *picture-text sequencing* principle, which postulates that, if a written text and a picture cannot be presented simultaneously, it is better to present the picture before the text than the other way round; the s*tructure-mapping* principle, stating that, if a subject matter can be visualized by different pictures in different ways that are informationally equivalent, it is better to use a picture with the form of visualization that is more appropriate to solve future tasks; the *general redundancy* principle, that suggests not combining texts and pictures if learners have sufficient prior knowledge and cognitive abilities to construct a mental model from one source of information; the *control-of-processing* principle, supporting the claim that, if a static picture is combined with text, the text is difficult to understand, and learning time is not limited, it is better to use written text rather than spoken text.

### Naïve conceptions about instructional tools

The features of the instructional devices are important, but we have to keep in mind that, in order to implement multimedia learning efficiently, attention has to be paid also to teachers' and students' beliefs about the cognitive processes activated by the educational tools to be employed (Antonietti and Colombo, 2008). Such beliefs—as noted by Veenman et al. (2006)—can be either correct or wrong. For instance, a teacher might assume that pupils can understand some subjects better if verbal descriptions are accompanied with multi-cultured and realistic photos which can increase the learners' curiosity and interest and so he/she might choose a textbook including such kind of visual materials, which actually fail to match Mayer's coherence principle. As another example, a student who has to learn a textbook page where words and graphics are simultaneously displayed in an interconnected way might think that it is preferable to read the whole set of sentences reported on the page first and then to look at the graphics, instead of shifting attention repeatedly from each sentence to the corresponding graphic (a process which that learner assumes to be too demanding), thus missing the advantages coming from the contiguity principle. Hence, it seems to be crucial that trainers and trainees share relevant beliefs about what is the optimal way to manage educational multimedia presentations (Kluwe, 1982; Brown, 1987; Nelson et al., 1998).

It is well-recognized that people's opinions about the instructional tools are not isolated, idiosyncratic beliefs, but rather they are usually coherently organized in well-defined conceptions which can influence the entire learning process, especially the standards that are set up as goals to be achieved, as well as the cognitive operations to be carried out to perform the task at hand (Bråten and Stromso, 2006; Bromme et al., 2010; Efklides, 2011). Such conceptions in fact act as an “apprehension structure,” which anticipates the task to be executed and helps learners to adapt themselves to the perceived characteristics of the task by metacognitively calibrating to them (Wilson and Bai, 2010). Various studies have shown that students with adequate beliefs about the instructional tools to be employed and the underlying mental processes usually achieve better learning results (Veenman et al., 1994, 2002; Antonietti et al., 2000; Crosier et al., 2000; Richardson et al., 2002; Stricker et al., 2004; Aydin and Ubuz, 2010; Fayena-Tawil et al., 2011).

### Naïve conceptions of multimedia learning

Students appear to be able to conceptualize benefits of using successful instructional technology which is supported by an adequate pedagogical approach (Mouza and Bell, 2001), such as when technology is implemented according to collaborative learning principles (Wilson and Whitelock, 1998). Students can also judge the quality of instructional materials not by superficial features, but by cognitive aspects. For example, students are able to judge the level of interaction and the amount of feedback offered when working online (Hackman and Walker, 1995). They can perceive, in a Web based training environment, clear and consistent differences between the best courses and the worst, basing their evaluation mostly on both fun and test scores (Hassett et al., 2003).

As multimedia devices are more specifically concerned, it has been shown (Antonietti and Giorgetti, 2004, 2006; Antonietti et al., 2008; Jacobson et al., 2010) that students and teachers are able to identify a large number of non-trivial opportunities from multimedia learning and possess well-defined and internally-articulated beliefs about the cognitive processes which are involved.

Naïve conceptions about learning in multimedia environments appears to influence students' performance. Liu et al. (2004) reported that learning outcomes are linked to students' beliefs about the distinctive features of educational tools. The efficiency of the learning process is associated with being aware of the actual potentialities of such tools, more precisely with the cognitive strategies that they allow students to apply to face the learning tasks. Jones (2001) maintained that students' attitudes and beliefs can reinforce the strength of visual annotations for gaining new knowledge from auditory text, thus promoting a more thorough processing of the passages and the usefulness of interaction with annotations. Huet et al. (2011) described some relationships between student's beliefs about help-seeking devices included in an interactive Web-based multimedia device and actual performance. Finally, the opportunity to exert control over learning in multimedia environments is beneficial if students have a high level of self-regulatory abilities (Scheiter and Gerjets, 2007) and relevant beliefs about the skills which are involved (Greene et al., 2010).

### The common sense theory of multimedia learning

Naïve conceptions as those mentioned previously are close to the «common sense theory» of multimedia, which refers to the beliefs that the general population (made up of non-experts in the specific field) shares concerning the properties, uses, advantages and disadvantages of multimedia (Mayer, 2001, 2005). The common sense theory, according to Mayer, should be opposite to his assumptions. Such a theory considers the human mind as a passive information elaboration system which relies on a single, unlimited processing channel. In Mayer's (2001) opinion, the common sense notion is that the main goal of multimedia instructional tools is to present information, so corresponding to an information-delivery theory. According to this view, if information is presented in the form of words, then presenting the same information in pictures adds nothing to learning.

## Aims

Mayer (2001) claimed that non-experts have inadequate conceptions regarding multimedia learning. Basing his conclusions on his survey concerning how multimedia devices (and, more precisely, combinations of texts and pictures in textbooks) are used, he inferred that his cognitive principles regarding multimedia learning are not shared by the general population (Mayer, 1993). From his analysis of textbooks (Levin and Mayer, 1993), Mayer concluded that writers and illustrators do not maximize the power of graphics to enhance multimedia learning (Mayer, 2001). This can be due—Mayer argued—to an ineffective underlying naïve theory, probably close to the informational-delivery theory. Moreover, Clark and Feldon (2005) explored people's questionable beliefs, especially concerning expectations of multimedia learning, matching them with experimental findings. They showed that commonly held principles are not supported by research.

Might it be that persons' naïve conception, in contrast with Mayer's claims, do not actually diverge from his theory? Mayer never tested his conjectures about the common sense theory, focusing his research on the experimentally-tested validity of his principles. The purpose of the present study is to address this topic.

Starting from the assumption that, albeit at different levels, all people have at the least some preconceived notions regarding the motives which induce designers of multimedia educational tools to combine pictures and texts in a given way and that they are able to verbalize and justify such ideas, an interview study was designed. We can presume that nowadays people are largely exposed to a variety of multimedia artifacts, not only in their professional domain. Moreover they actually use many such artifacts and, more importantly, they can select the preferred item within a category of multimedia tools, and thus it is likely that they base their choices on specific beliefs about the quality of the item. Finally, mass-media communication spreads the idea that we live in an “image society,” so leading people to reflect upon the meaning of this message.

The aim was to investigate the conceptions shared by different subsamples about the functions and the usefulness of different typologies of illustrations. In this framework we decided to explore multimedia products as intended by traditional literacy, that is, a product, which combines texts and pictures (Houston, 1988).

The main goal of this study, therefore, is to assess naïve conceptions about multimedia learning. The term «naïve» is used here not to say that these conceptions are intrinsically inadequate since they do not correspond to the specialists' claims. Beliefs about multimedia learning are labeled as «naïve» since they are developed by people who do not have a specific competence about the cognitive mechanisms underlying the comprehension processes activated by text-picture combinations. Different levels of naivety can be identified. Laypersons who have no current experience with multimedia learning can be placed at the lowest level. These persons are not intentionally exposed to multimedia presentations with training aims and so they are not usually induced to pay attention to the features of educational multimedia instruments. Their beliefs about such instruments are based not on personal engagement with multimedia presentations as learners or instructors, but on the indirect knowledge that they can derive by looking at them (in bookshops, libraries, advertisements) or by observing other people (their own sons, for instance) using them. We can place students and teachers at an intermediate level. Both are using multimedia textbooks as learning tools and their concrete experience with them can lead these categories of individuals to became aware of the potentialities (by realizing when, how, and why learning is facilitated) and limits (by perceiving the difficulties they encounter, for instance) of the different kinds of text-picture combinations, so developing deeper beliefs about them. Illustrators can be placed at the highest level. They do not possess an explicit, scientifically-grounded expertise about the cognitive mechanisms underlying multimedia learning, but the job of devising pictures to be associated with texts should induce them to reflect about what kinds of images are relevant and what are not, where to locate the images, their optimal size, colors, and so on.

The study attempted to investigate three main issues.

1. The features of naïve conception about multimedia learning. As far as this issue is concerned, the aim is descriptive and so no specific hypothesis was built. As yet the lack of empirical data prevents us expecting what people actually believe about the mental processes they think are activated when someone is exposed to a given combination of texts and pictures. Attention is focused on two specific aspects:
  - The *cognitive functions* attributed to different kinds of pictures, namely, the beliefs about the role—expressed in terms of mental processes (e.g., enjoying, visualizing, connecting, understanding notions)—played by pictures.
  - The *perceived utility* of different pictures, that is, the beliefs about the match/mismatch between the actual and the optimal mental processes activated by pictures.
2. The internal coherence of the above mentioned conception is investigated. Since literature supports the notion that even naïve people have well-defined and stable beliefs about mental processes involved in learning tasks, we hypothesize that consistent associations among participants' responses given to different requests concerning the same issue and relationships among different aspects of the conceptions will emerge.
3. Differences among subsamples concerning the conception in question. We hypothesize both that higher levels of expertise lead people to identify the mental processes involved in multimedia learning in a more adequate way and that a specific expertise in the target field (the use of illustrations for educational purposes) influences how individuals represent such processes, by stressing the aspects which are more linked to their profession (e.g., teachers should emphasize how understanding can be improved by pictures).

## Methods

### The interview

It was decided to use an interview, rather than a questionnaire (as in some previous studies), to investigate people's beliefs, as an interview allowed us to go into more detail on each single topic, without affecting the respondents' answers. Interviews provide in-depth information about a particular research issue or question and allow us to analyse the responses using a method which provides us with a “big picture” that transcends any one single bit of data. This is possible since a qualitative research interview seeks to cover both a factual and a meaning level (Kvale, 1996), and this appeared to be particularly appropriate for our exploratory study. Questionnaires seemed to be less suitable for our aims, since they may influence respondents' answers, as, by their nature, they organize the target topics *a priori*. Moreover, questionnaires only ask for agreement/disagreement on general statements. Since it is unlikely that people state their beliefs as general statements, it is easier for them to express such beliefs with reference to specific situations—as provided by our interview—rather than as preset, generalized statements. Thus, we tried to encourage people to declare their opinions freely and on specific and concrete cases. Furthermore, as the interview was based on specific pictures taken from actual textbooks, the interview format was deemed likely to secure more valid data because of the greater ecological validity of the employed materials. Considering again the fact that beliefs are often not available as explicit abstract conceptualizations, our interview did not require participants to give only verbal answers, but also asked them to respond through practical actions, such as selecting pictures and placing them in such a way as to create an original product.

Each participant was asked to answer a series of questions within the same interview, which was structured according to the criteria explained below. Materials used during the interview were: (1) a booklet, which included a selection of textbook pages; (2) the materials for the Perfect Book; (3) a filing record.

#### Booklet

To build his functional classification, Mayer (1993) considered 6 sixth-grade science textbooks approved for adoption in California. To assess the exact typology of each image, Mayer referred to Levin's (1989) functional classification. In accordance with the author's cognitive theory, the category the pictures belong to may elicit different cognitive processes. More precisely:

1. *Decorative* pictures: they are not directly relevant to the text and should not have any effect on any cognitive process.
2. *Representational* pictures: they portray one element that is mentioned in the text and should influence the process of selection.
3. *Organizational* pictures: they represent the relations among elements in the text and should influence the process of selection and organization.
4. *Explanative* pictures: they explain how something (e.g., a system, a process, a mechanism) works and should influence the process of selection, organization, and integration.

To build the research protocol we decided to focus on primary schools, where the use of illustrations to match text is greater (Colombo et al., 2009), so as to be suitable for the still developing skills of reading texts in children. Unlike Mayer (1993), we did not focus only on one grade or age group, but considered textbooks related to all primary school grades (that is, according to the Italian school system, from the first to the fifth grade). Moreover, we also decided to examine not a single discipline, but the full range of subjects introduced in primary school, considering the fact that people might have subject-specific conceptions about the use of illustrations.

To select the textbooks to analyse, reference was made to the official classification^1^ of the books most frequently chosen by Italian teachers during the 2004/2005 school year. Data for this study has been collected during 2005/2006 academic year—so this information was the more appropriate to use for the book selection. As in Italy it is the teacher of each individual school who actually chooses the texts to be employed for the different grades each year, such a list should in fact reflect the true preferences of Italian teachers. Textbooks for every single class of primary school were taken into account. For each textbook, the space devoted respectively to pictures and texts was quantified, while all the illustrations were classified^2^.

The analysis of pictures was carried out on the basis of Mayer's classification (1993) and, hence, the iconic material was divided into Decorative (D), Representational (R), Organizational (O), and Explanative (E) illustrations. During an initial pilot analysis, however, it was felt that some categories needed to be specified further. Specifically, while examining Decorative and Representational pictures, differences were found within the same category. Thus, it was decided to split the Decorative category into two distinct subcategories: first-level (D1), with no reference at all to the text, and second-level (D2), where analogies or connections with the text are present, albeit weak. Examples of D1 illustrations include all those elements that decorate the page, such as page frames and borders which often fill the empty spaces. D2 illustrations, on the other hand, are slightly more appropriate images, such as the picture of children playing in the woods placed close to a text referring to photosynthesis. While this picture does not represent any part of the text, a weak reference to the general topic can be found. The distinction outlined for Representational pictures was as follows: first-level Representational pictures (R1), which actually represent parts of the text, and second-level Representational pictures (R2), which represent additional elements, that is, elements that somehow enrich or integrate the text. An example of R1 pictures may be recognized in an illustrated tale: each picture representing a scene of the story is a first-level Representational picture, since it shows a part of the text. An R2 picture, on the other hand, is an illustration that replaces the text or that adds something to the text, such as the photo or drawing of different types of leaves, with a detailed caption explaining the differences among them, placed in close proximity to a text explaining vegetation, which mentions trees and leaves but does not provide details on the different types of leaves (see Table 1).

**Table 1:** **Examples of pictures for each category (all illustrations refer to the same text which describes the photosynthesis)**.

**Types of Illustration**	**Example**
***Decorative I level***	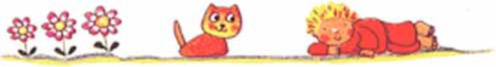
Decorative Image-no reference to the text	
***Decorative II level***	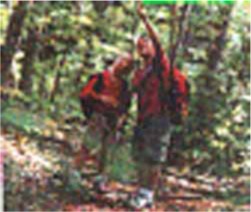
Decorative Image-analogies or connections with the text are present, albeit weak	
***Representational I level***	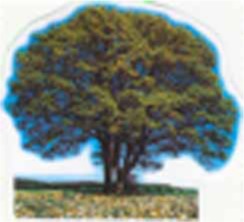
Represent parts of the text	
***Representational II level***	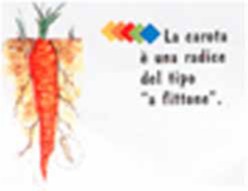
Represent additional elements that enrich or integrate the text	
***Organizational***	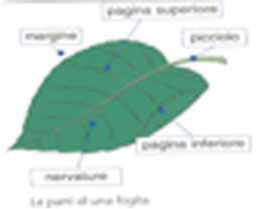
Represent the relations among elements in the text	
***Explanative***	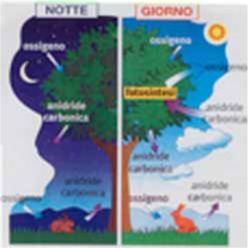
Explain how something works	

The pilot categorization of the illustrations led us to devise a structured system of classification—based on the distinction among D1, D2, R1, R2, O, and E pictures—which included a clear operational definition of each kind of image. Such a definition was expressed in terms of conditional clauses (e.g., “if the picture depicts an item mentioned in the corresponding text, then exclude D1 and D2 categories and consider the other categories”) so as to produce a sort of decision tree which avoids ambiguity and hence allows encoders to reach a unanimous judgment. It is worth noting that the classification system we devised is metacognitive in its nature: the different types of pictures were defined according to the mental process they involve, which is the basis of its function in learning (Table 2).

**Table 2:**
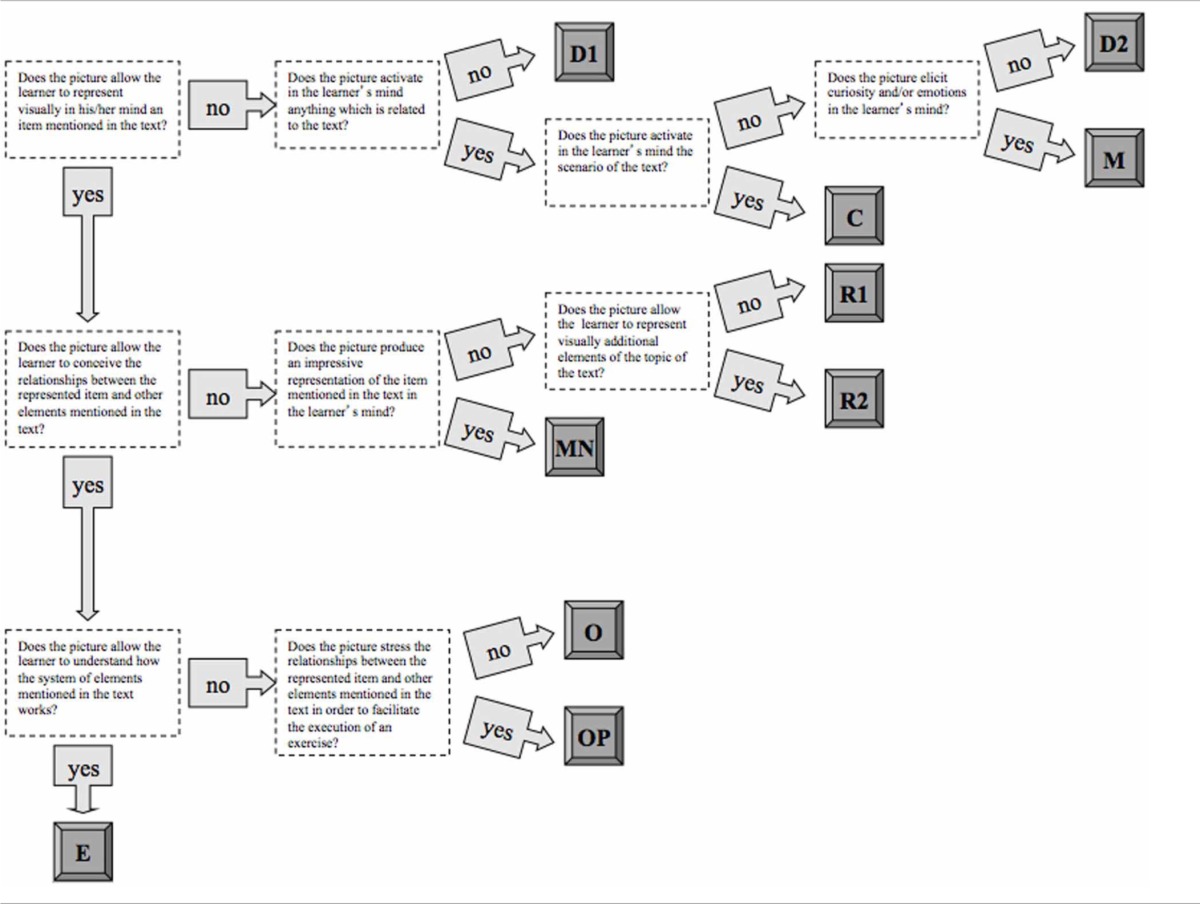
**Taxonomy of the categories of pictures according to their cognitive functions**.

After having devised the classification system, the actual categorization took place. All pictures were coded by two previously trained judges and assigned to the corresponding categories. The agreement between the two judges was 100%.

After analysing the textbooks, we devised the actual booklet to be used in the interview, which consisted of 36 pages, derived from all the textbooks analysed. The page selection criteria were:

- *Representativeness*:

1. For each grade, texts appearing in the first, second, and third place in the official classification of the textbooks approved for use in the Italian school system were considered.
2. For each textbook type (single text for grades 1–3 and reading and disciplinary textbooks for grades 4–5), pages from textbooks for all grades were included.
3. For each topic, the number of pages devoted to it (geography, history, maths, arts, language, science) were roughly equivalent.
4. For each grade, at least two examples of each category were included (D1, D2, R1, R2, O, and E).

- *Economy of space*: pages distinguished by the simultaneous presence of pictures belonging to different categories^3^ were favored.
- *Meaningfulness*: pictures clearly consistent or inconsistent with Mayer's principle (specifically, the spatial contiguity principle, since the other principles were not applicable to our specific material. Mayer's principles were devised for multimedia computer-supported presentations and would hardly have been transferable as a whole to text pages) were inserted in the booklet. To determine whether an illustration was or was not consistent with Mayer's spatial contiguity principle, Mayer's definition was taken into account: the pictures selected by the researchers were analysed to see if they met all the conditions considered as distinctive points, such as the proximity of one or more illustrations to the corresponding test sections and the relevance of those illustrations to the text being referred to. If they met all the conditions mentioned in Mayer's principle, they were considered to be consistent with the principle. If those points were not met, the picture was not considered to be consistent. The agreement between the two researchers who checked the coherence/incoherence with the contiguity principle was 98%. Ambiguous cases were evaluated and solved by the judges.

Overall, 95 categorized images in the booklet were reported and divided among functional categories: 10 D1, 13 D2, 14 R1, 18 R2, 22 O, and 19 E pictures. The number of pictures for the different categories was not the same for each subcategory, as the total number of selected illustrations was proportional to the number of pictures in each category in the analysed textbooks. It is also important to note that D1 and D2 pictures and R1 and R2 pictures were each considered (for the total amount) as a single category. That is to say, we considered Decorative pictures as a whole (bringing the number of Decorative pictures to a total of 23) and Representational pictures as a whole (bringing the number of Representational pictures to a total of 32). The sequence of the selected pictures in the booklet was randomly set.

It is important to highlight that all the pages selected to compose the booklet were always full pages, which were not related to any other pages, but were autonomous in their meaning. This selection had been made so as not to influence interviewees' performance or interpretation.

#### Materials for the perfect book

The materials were as follows:

- Two white cards (size slightly above A4). They represented the hypothetical pages of an ideal book;
- A brief didactic text relevant to primary school (telling about vegetation and photosynthesis). The text was composed by elaborating upon an actual text found in the examined textbooks;
- Fourteen illustrations belonging to different categories, specifically: 2 D1, 2 D2, 2 R1, 2 R2, 3 O, and 3 E pictures.

#### The filing record

It was composed of three parts.

- A section for collecting general information about the participant (gender, age, occupation/study course).
- Grid of picture analysis. The interviewee was presented with the booklet described above (see point 1). Each page had previously been categorized according to the criteria given. The interviewer, on his/her part, had a grid in which the different pictures for each page of the booklet were labeled by means of abbreviations and which provided space to record the interviewee's answers (see below).
- The record of the most- and least-useful pictures and of the Perfect Book. At the end of the protocol there was room to record the answers to these last two requests (see below).

### Sample

One of the goals of the study was to identify the naïve theories of people who are differently experienced with the text-illustrations relationship. As stated previously, the targets of the study were illustrators of children's literature, teachers, and students; that is, the kinds of persons who, respectively, create, choose, and use textbooks. They are experienced in the field of multimedia (with the basic meaning of artifacts where text and pictures are used together) but are not trained in the corresponding specific cognitive theories. A group of adults with no formal experience in multimedia use constituted the control subsample.

Four sub-samples of participants were created, each one corresponding to a different category of subjects. The interviewees were all volunteers who had been contacted personally by the researchers and asked about their interest in the research topic and their willingness to join the study. They were neither paid nor given any academic credits for their participation.

*Illustrators* (*N* = 16; men = 4, women = 12; age range = 21–62 years, mean age = 39.25 years, *SD* = 12.52): this subsample included people with work experience in the development of multimedia products. The majority of the participants in this subsample were illustrators specializing in (text) books for children. Graphic designers (who however had a basic training in the illustration field), who generally possess a broader experience in the field of multimedia products (such as Web-sites or didactic software), were annexed to the group to balance the sample, which would otherwise have been comprised solely of illustrators used to working mainly with paper-and-pencil tools and only occasionally with technological tools. The illustrators, listed in the annual of the members of the Italian National Illustrator Society, were recruited by means of a letter in which they were asked if they would be interested in joining the investigation.

*Teachers* (*N* = 18; men = 4, women = 14; age range = 22–53 years, mean age = 37.22 years, *SD* = 11.04): this subsample was made up of primary school teachers. In addition, if they proved to have more than 1 year of teaching experience within school institutions, students attending the last year of the degree to become Primary school teachers were also included in this sub-sample. These teachers, employed in Italian private and public primary schools, were recruited by means of a letter asking them about their willingness to join the study. Students were selected among those who subscribed to a newsletter of a service of learning psychology which offers information and counseling to teachers of all grades.

*Students* (*N* = 18; men = 7, women = 11; age range = 19–32 years, mean age = 22.83 years, *SD* = 3.17): this category comprised university students attending different faculties in universities located in Milan and was evenly divided between students of science and of arts courses. This specific sub-sample was considered because, in his research, Mayer had used mostly university students as participants and had based his assumptions about the common-sense theory on data collected working with them. Thus it was considered interesting and appropriate to examine and compare naïve theories also from this particular group. Moreover, learners appear to make better decisions than teachers or instructional designers because they have a bigger stake in the educational outcome and intimate knowledge of their learning preferences (Niemiec et al., 1996). The students were recruited by means of a notice placed on noticeboards in the university campuses. The selected students had no previous knowledge either of learning or cognitive psychology, or of multimedia learning.

*(Control) adults* (*N* = 16; men = 8, women = 8; age range = 22–61 years, mean age = 32.94 years, *SD* = 11.20): this group included adults with jobs that do not require specific multimedia competence or experience with reference to the multimedia material employed in the present study. More precisely, these jobs were: office worker (8), maintenance man (1), physician (1), nurse (1), engineer (5). They were recruited by means of a notice placed on noticeboards for the administrative and technical staff of the university campuses.

### Procedure

The procedure followed by the researcher while administering the interview was structured as follows. Each participant was individually interviewed. Before starting the interview, a brief explanation about the goals of the study was given to the participant. It was explained that the study was aimed at investigating the text-picture relationship in primary school textbooks and that researchers were interested in exploring participants' perceptions of this relationship. Interviewees were told explicitly that the questions were not aimed at any sort of evaluation of the participants and that, therefore, no answers could be considered either right or wrong. Then the interview procedure was briefly explained.

Afterwards, as reported in the protocol description, data aimed at describing the participant were recorded. After these preliminary phases, three requests were made.

#### First request

The interviewer presented the participant with the booklet. Before beginning the interview, he/she specified to the participants that in the study «pictures» referred to both illustrations and graphical elements such as schemata. The interviewee was then asked to examine the illustrations on each page and to answer two questions:

«What, in your opinion, is the purpose of each different picture?»

«Assign, for each illustration, a score between −2 and +2, referring to the level of usefulness/uselessness you ascribe to the picture»^4^.

The first question was aimed at outlining the subjects' naïve conceptions concerning the functions of pictures expressed in terms of mental processes involved during learning. The second question was meant to highlight people's beliefs concerning the usefulness of the different typologies of illustrations by inducing them to match the mental process actually activated by pictures to the alleged optimal process.

#### Second request

In order to understand which naïve theories concerning the efficacy of text-picture combination people tend to share, the interviewees were asked to indicate which illustrations they considered to be the most and the least useful, after they had evaluated all the booklet's pages and seen the illustrations during the first part of the interview. Such a request involved a form of comparison with the naïve conception resulting from the first request. Forcing a direct comparison between pictures and leading the respondents to divide the images into two distinct categories, we tried to confirm, or disconfirm, the more general evaluation given in the first phase. In the second part of the first request, participants were asked to rate each illustration singly, stating the level of usefulness for each of them. This second request was more general (asking the interviewees to give an overall evaluation of the usefulness/uselessness of the pictures). It was aimed at integrating the first evaluation by adding data coming from a more general evaluation. This second assessment was imagined to be more focused on general characteristics of the mental processes elicited by illustrations and less on the specific processes elicited by single pictures.

#### Third request

The interviewee was asked to build his/her Perfect Book, using all or some of the provided material mentioned above. The text on photosynthesis was divided into two pages and the interviewee was free to place both the textual and visual information in the order and the amount he/she wished on the pages. The arrangement of illustrations on the page was also left to the interviewee, who was free to place the chosen pictures and the corresponding text where he/she believed them to be most useful, easily read, or best comprehended. The researcher then reproduced the final composition on the filing record. More precisely, he/she schematized the general structure (i.e., the arrangement of the elements) chosen by the participant and assigned a code (corresponding to the category to which each picture belonged and a progressive number for each portion of text) to each picture and piece of text chosen by the interviewee. The goal of this task was to highlight the possible coherence of naïve theories with Mayer's spatial contiguity principle, as well as provide a comparison with the statements of the first and second requests in order to assess the internal consistency of the respondents' beliefs.

The following schema shows the correspondence between the research issues and the steps of the interview (X indicates that the question was considered in that phase of the interview).

**Table:** 

**Research focus**	**Requests to interviewees**		
	**State the aim of each picture (first request)**	**State the most/least useful picture typology (second request)**	**Build the Perfect Book (third request)**
(1a). Description of the naïve conception: functions of the pictures	X		
(1b). Description of the naïve conception: utility of the pictures		X	X
(2). Internal coherence of the naïve conception		X	X
(3). Differences among subsamples concerning naïve conception	X	X	X

Two researchers carried out the interviews. Both have been trained to ask the questions and to record the answers according to a standard procedure.

### Data analysis

#### Overall design of the analyses carried out

The collected data were presented in an order which mirrors the structure of the filing record explained previously and were analysed according to the three main research issues mentioned at the end of the section “Objectives of the study” so as to answer the specific questions listed below:

1. Features of the conceptions about multimedia learning
  (1a) *Cognitive functions* attributed to pictures
    - What mental functions do interviewees ascribe to different pictures?
    - Is the function classification identified by the participants similar to Mayer's?
  (1b) *Perceived utility* of pictures
    - Do utility scores differ according to pictures' cognitive functions?
    - Which pictures are perceived as the most and least useful?
    - Do the utility scores assigned by the participants match Mayer's model?
2. Internal coherence of the conceptions about multimedia learning
  - Is it possible to identify a general coherence among usefulness scores and between the general scores attributed to pictures and the composition of the Perfect Book?
3. Differences among sub-samples concerning naïve conception about multimedia learning
  - Does any significant difference emerge among sub-samples in function attribution?
  - Does any significant difference emerge among sub-samples in utility scores?

#### Data encoding

Opinions concerning the purpose of pictures (first request) were coded into 11 functional categories. The system of categories devised by Mayer (1993), with the extensions and modifications imposed in our study, appeared to be a good point from which to begin classifying the respondents' beliefs about the function of each picture in promoting pupils' learning. However, an initial examination of the interviewees' answers suggested that functions not included or foreseen in Mayer's system had emerged. Thus, some additional categories were needed. Distinctive characteristics expressed by the interviewees, and not corresponding to the elements of the functional categories previously considered, were registered, examined, and organized into four new categories that had not been considered by Mayer. Categories 1–7 correspond to our original classification derived from Mayer's own classification, whereas categories 8–11 are based on the additional data gathered regarding participants' beliefs^5^.

The four additional categories are as follows:

- *Contextualizing* (C): pictures which, at various levels, introduce the main topic. These help the student to link the text content to his/her everyday life, or to make some unknown aspect of experience more familiar (for example, the photo of a girl eating an ice-cream next to a text explaining the taste buds). Illustrations that characterize or identify a specific subject or activity can also be related to this category.
- *Motivational* (M): illustrations which excite curiosity or elicit emotions and attract the student's attention so as to induce him/her to read the text or perform the required activity. Images which stimulate the identification of the student, involving him/her from an emotional point of view are also included in this category (for example, cartoon characters or cartoons put beside a maths exercise or explanation).
- *Operational* (OP): pictures which are an essential and needful part of an exercise or activity. In this case it is not the iconic typology that has to be taken into account, but rather the final purpose of the illustration within the context of a specific activity (for example, illustrations of the single parts of a practical activity to be performed by students). The interviewee, when referring to this category, stressed its relevance to the activity of which it is a part.
- *Mnemonic* (MN): pictures which allow students to activate memorization strategies by using mental visualization (or more general visual strategies) or by using concrete objects as an aid in maths exercises.

This system of categories—as well the distinctions between D1, D2, R1, R2, O, and E pictures—was converted into a set of unambiguous operational definitions, expressed through conditional clauses, hierarchically organized in a decision tree so as to allows encoders to apply the same classification criteria.

The coding procedure was carried out by two independent judges, who were trained to apply the above mentioned categories to the interviewee's answers. All responses had been read by the two judges and assigned to different categories. Responses were then re-examined and the assignment to the specific categories had to be confirmed. The agreement between the two judges was 98%. Ambiguous cases were evaluated by the two judges together in order to reach a unanimous decision.

## Results

### Features of the conceptions about multimedia learning

#### Pictures' functions

Analyses were carried out separately considering textbooks of each grade of the primary school and the distinction between reading and disciplinary textbooks. Since the pictures that emerged overlapped, we collapsed data and so we will present results concerning the whole set of textbooks considered without distinguishing among grades and kinds of books.

Table 3 reports the frequencies and percentages of participants' function attribution. The types of functions ascribed to the illustrations by the interviewees were compared with Mayer's classification in order to highlight correspondences and discrepancies. The columns show how the pictures were grouped according to Mayer's classification and the percentages of these groupings, whereas the rows show the same pictures grouped according to interviewees' attributed functions. For example, 59 pictures classified as D1 according to Mayer's classification were considered as Decorative by participants.

**Table 3:**
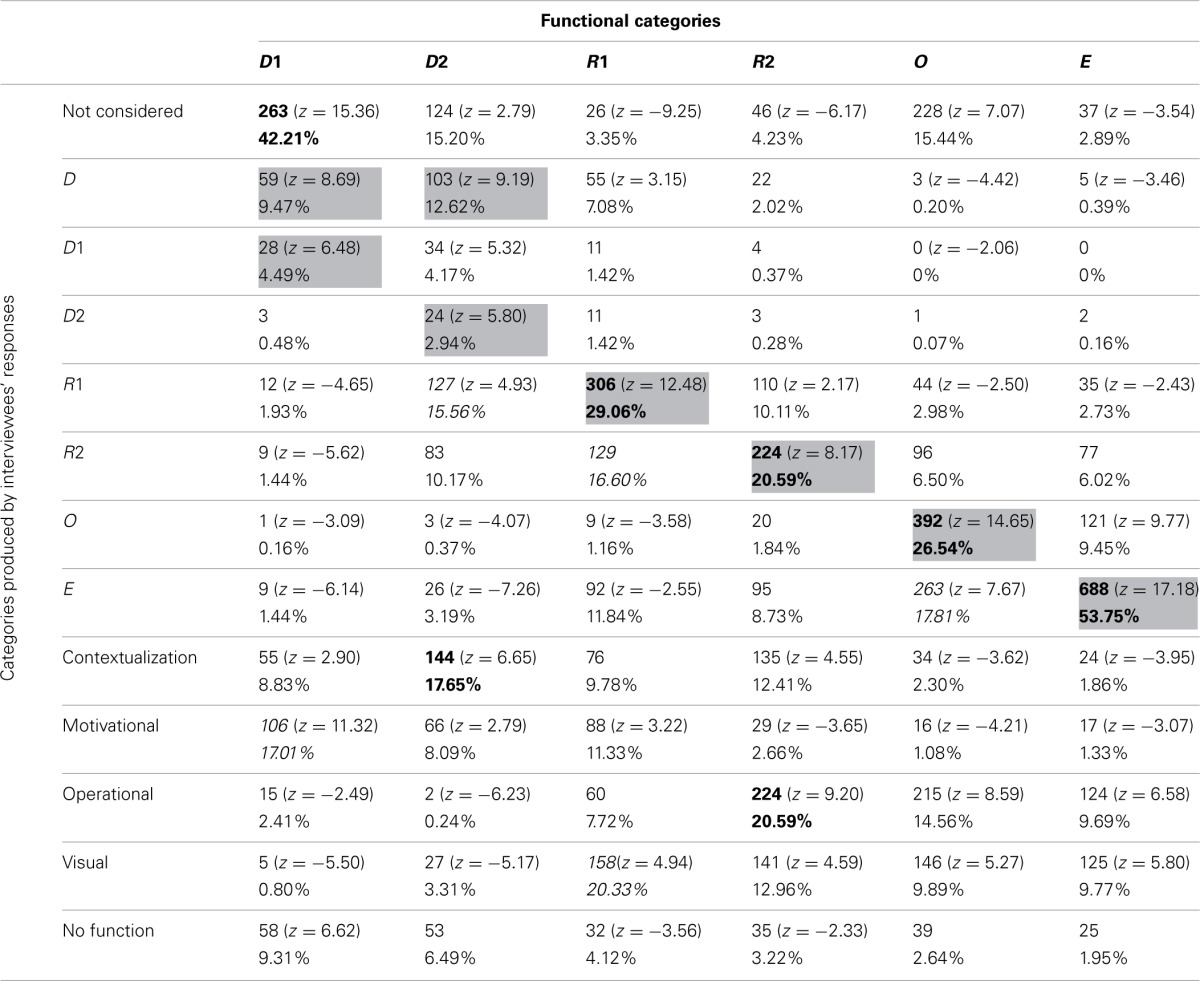
**Frequencies and percentages of function attributions**.

z values between 1.65 and 2.32: p < 0.05; z values between 2.33 and 2.57: p < 0.01; z values between 2.58 and 3.10: p < 0.005; z valueas equal or higher than 3.11: p < 0.001.

The gray cells in the table denote where the majority of choices would be grouped if participants' categories completely corresponded with Mayer's classification. The bold emphasis indicates where the highest frequencies occurred. *Z*-value tells us how many standard deviations above or below the mean our value *x* is: positive *z*-values are above the mean, whereas negative *z*-values are below the mean.

A log-linear analysis was performed on the frequency data in Table 3. Given the specific data and the number of variables, we decided to test the data using a log-linear analysis, since One-Way ANOVAs and chi-square tests would not have allowed us to consider the interactions among the different variables. The analysis resulted in a hierarchical log-linear model, where the interaction between the variables was significant [χ^2^_(60)_ = 5104.92, *p* < 0.001]. As it appears from the data reported in Table 3, R1, E, and O were categories where a good correspondence with Mayer's functional classification can be found. The R2 pictures were quite correctly detected by many participants, as were the OP ones. This can probably be ascribed to the massive use of R2 pictures (those which, as explained above, integrate or stand in for the text) to complete, describe, or enrich exercises or practical applications.

The remaining categories (D1 and D2) were classified by participants in a different way. More precisely, D1 pictures were, on the whole, not considered by participants. Those who actually considered them did not see them as decorative, but ascribed to them a motivational function. D2 pictures were more frequently considered by participants, who, however, did not seem to have a clear idea of their function. The majority (which, nonetheless, coincides with only 17.65% of the answers) saw them as C pictures, most likely because several D2 pictures were photos that emphasized the link with concrete experience. Many participants saw these pictures also as Representative because of a weak connection with the content of the corresponding text.

R1 pictures were mostly ascribed to the MN category. This attribution is due to the fact that many pictures related to this category were used to represent texts of mathematical problems. Therefore, they might appear to the participants as providing suitable mental strategies to better comprehend and solve problems. It is worthwhile noting that 16.6% of interviewees classified the R1 pictures as R2 ones, implying that participants tended to attribute a greater function to the role of the picture than it actually had.

#### Pictures' utility

*Utility scores:* For each illustration, participants were asked to assign a score between −2 and +2, according to their perception of the picture's level of usefulness/uselessness. To analyse the mean utility scores assigned to the different picture categories, the scores were converted into a 1 to 5-point scale. The overall scores have been computed and the mean and standard deviation for each category have been calculated (Table 4). The ANOVA (computed by considering the categories as the independent variable) showed that utility scores differed significantly among categories, *F*_(5, 402)_ = 165.51; *p* < 0.0001. *Post-hoc* Turkey tests showed that, excepting the R1 and R2 pictures, all the differences among picture categories were statistically significant. This led us to conclude that interviewees were able to discriminate among picture categories while rating them.

**Table 4:** **Means and *SD*s of utility scores attributed to different picture categories**.

**Category**	**Mean**	***SD***
*D*1	1.90	0.74
*D*2	3.01	0.56
*R*1	3.96	0.46
*R*2	3.99	0.49
*O*	3.66	0.57
*E*	4.26	0.52

From data reported in Table 4, it can be seen that D1 pictures were considered to be less useful, whereas the E pictures were perceived as being the most useful. D1 pictures had the highest standard deviation: this can be explained by the fact that most subjects tended either to ignore such pictures or to ascribe them a positive score when actually considering them. D2 pictures collected a much higher utility score compared to D1 pictures, maybe because our interviewees focused more on the possible connection with the corresponding text. O pictures were rated lower than R pictures: maybe this lower rate was due to the fact that many O images, being similar to schemata and/or graphs, were not recognized as proper illustrations by many participants (actually O pictures had the highest SD after the D1 ones). Yet those who considered them rated them highly.

*Most and least useful images:* In the second phase of the interview participants were asked to indicate the pictures which they considered to be the most and least useful. Data resulting from the analysis of interviewees' responses seemed to be consistent with the utility scores assigned to each picture and described above. Considering this second request as a direct confirmation of the first request, we decided to focus on the participants' attribution of functions.

The pictures defined as the most useful (Table 5) are the E pictures. High percentages were also found for the O, C, and MN pictures. None of the interviewees designated the Decorative pictures as the most useful typology. Differences in the distribution of the illustrations mentioned as the most useful were significant [χ^2^_(8, *N* = 68)_ = 44.5, *p* < 0.001].

**Table 5:** **Frequency of pictures rated as most and least useful**.

**Most useful images**	**Frequency**	**Percentage**
No image	1	1.5
Representational 1	7	10.3
Representational 2	3	4.4
Organizational	10	14.7
Explicative	23	33.8
Contextualization	9	13.2
Emotional-motivational	4	5.9
Operational	4	5.9
Visual-mnemonical	7	10.3
Total	68	100.0
χ^2^_(8, *N* = 68)_ = 44.5, *p* < 0.001		
**Least useful images**	**Frequency**	**Percentage**
-Emotional-motivational^*^	1	1.5
-Contextualization^*^	1	1.5
-Explicative^*^	1	1.5
-Organizational^*^	1	1.5
-Representational 1^*^	5	7.4
No image	3	4.4
Decorative	16	23.5
Decorative 1	20	29.4
Decorative 2	3	4.4
Representational 1	4	5.9
Organizational	1	1.5
Contextualization	2	2.9
Emotional-motivational	1	1.5
Images with no function	9	13.2
Total	68	100.0
χ^2^_(13, *N* = 68)_ = 97.94, *p* < 0.001		

* In this table, categories preceded by the sign “-“ refer to the non-achievement of the function. For example, “- Representational 1” refers to a missed Representational picture of first level, namely, a Representational picture of first level which is not able to adequately represent the text it refers to.

The majority of the interviewees indicated the Decorative pictures as being the least useful (Table 5). Other categories mentioned in response to this question included pictures defined as “useless” from the outset and those not considered as fulfilling their function (e.g., decorative pictures which do not properly represent the text referred to) [χ^2^_(13, *N* = 68)_ = 97.94, *p* < 0.001]. Respondents did not select pictures categorized as E to answer this question.

### Internal coherence of naïve conception about multimedia learning

Almost all the participants (91.2%) used both pages to compose the Perfect Book. The number of images used was, for most interviewees, about half of the provided illustrations: 23.5% of the participants used 7 pictures out of the 15 total available, 17.6% used 6 pictures, and another 17.6% used 8 of the proposed images. Only 13.2% used 4 pictures, while 2.9% used 9. The main trend was to use 2 O (72.1%), 1 (41.2%) or 2 (50%) E and R (1 picture = 36.8%, 2 pictures = 39.7%) and one (30.9%) or no (41.2%) D illustration. All participants observed the spatial contiguity principle.

A strong coherence between the perceived utility among the three requests emerged and is summarized in Table 6. Considering the different requests, it was not possible to match answers and requests perfectly. All of the answers were split into three principal categories: High utility (corresponding to those pictures used most often to compose the Perfect Book, those pictures rated as most useful, and those categories that gained the highest mean of utility scores); Medium utility (corresponding to those pictures used less often than others to create the Perfect Book and those categories that gained a medium mean of utility scores); Low utility (corresponding to those pictures used the least to make up the Perfect Book, the pictures rated as least useful, and those categories that gained the lowest mean of utility scores).

**Table 6:** **Coherence among the three requests regarding picture utility**.

	**Perfect Book**	**Most useful pictures**	**Least useful pictures**	**Means of utility scores**
High utility	Organizational, Explanative	Organizational, Explanative		Organizational, Explicative
Medium utility	Representational			Representational first level, Representational second level
Low utility	Decorative		Decorative	Decorative first level, Decorative second level

What emerges is that there is not only a strong coherence among the three requests, but also a generalized model of picture utility, which mirrors Mayer's classification. The fact that all the participants spontaneously observed the spatial contiguity principle while composing the Perfect Book leads us to think that this principle, too, was implicitly perceived as valid for our sample.

### Differences among sub-samples concerning naïve conception about multimedia learning

#### Pictures' functions

Frequencies and percentages of category attribution for each group of participants are reported in Tables 7–10. Mayer's functional categories were compared with the categories ascribed by participants (as was previously done for the whole sample) to highlight correspondences and discrepancies between the two positions. Gray cells denote where the majority of the choices would be grouped, should participants' categories completely correspond with Mayer's categorization. Bold emphasis indicates where the majority is actually grouped.

**Table 7:**
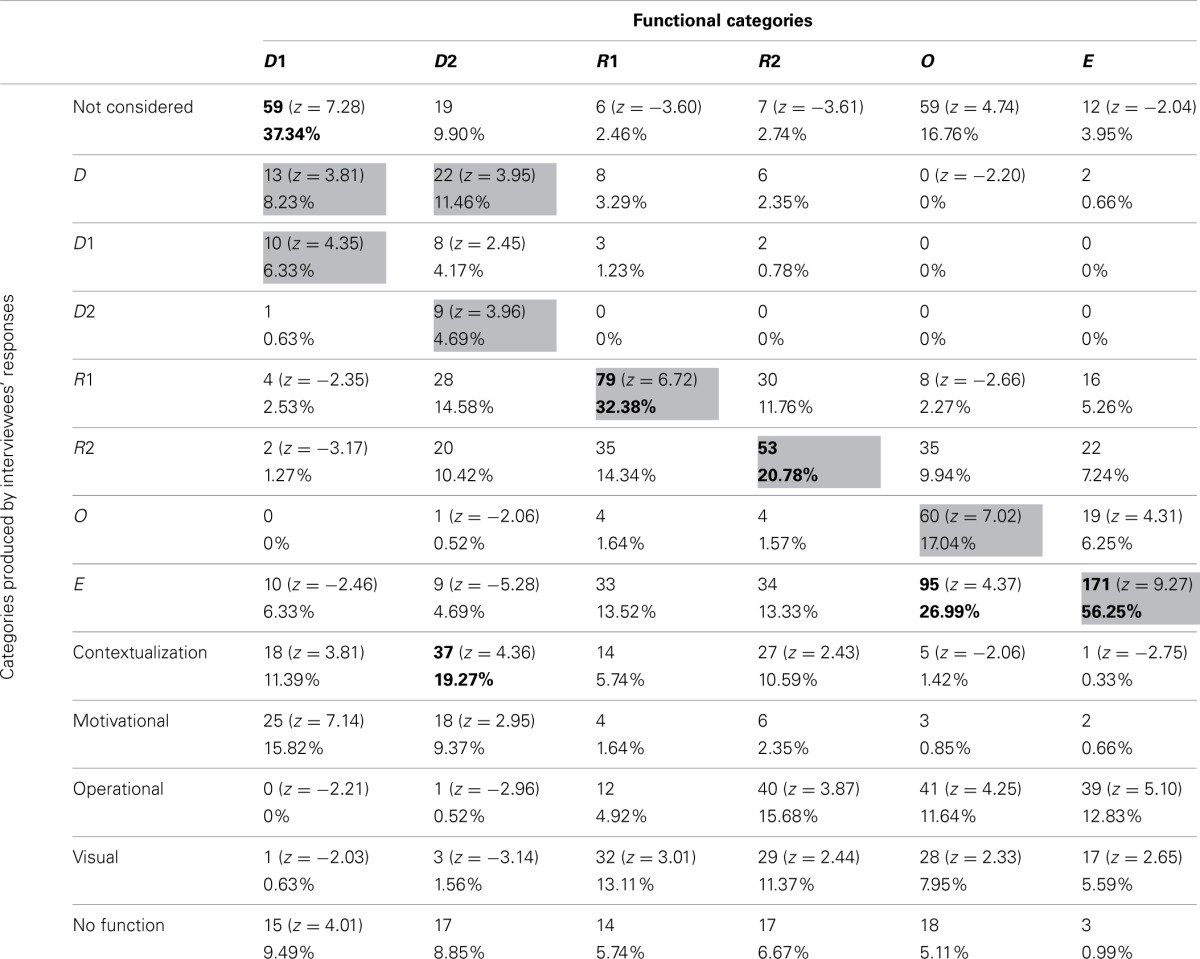
**Frequencies and percentages of category attribution for illustrators**.

A log-linear analysis was performed on the frequency data reported in the four tables. In all cases the analysis specified a hierarchical log-linear model, where the interaction between the variables was significant [Table 7: χ^2^_(60)_ = 1091.69, *p* < 0.001; Table 8: χ^2^_(60)_ = 1490.96, *p* < 0.001; Table 9: χ^2^_(60)_ = 1769.28, *p* < 0.001; Table 10: χ^2^_(60)_ = 1328.55, *p* < 0.001].

**Table 8:**
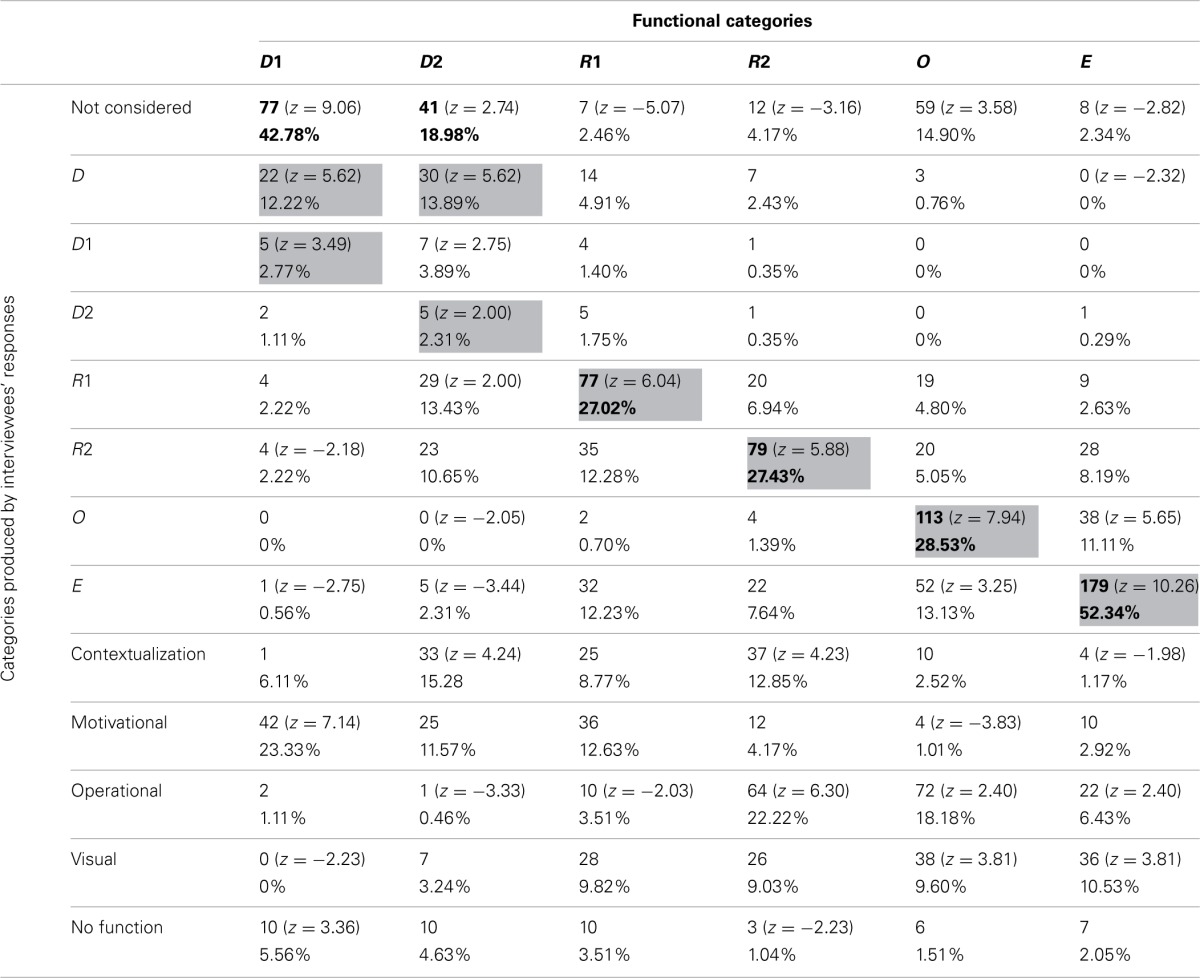
**Frequencies and percentages of category attribution for teachers**.

**Table 9:**
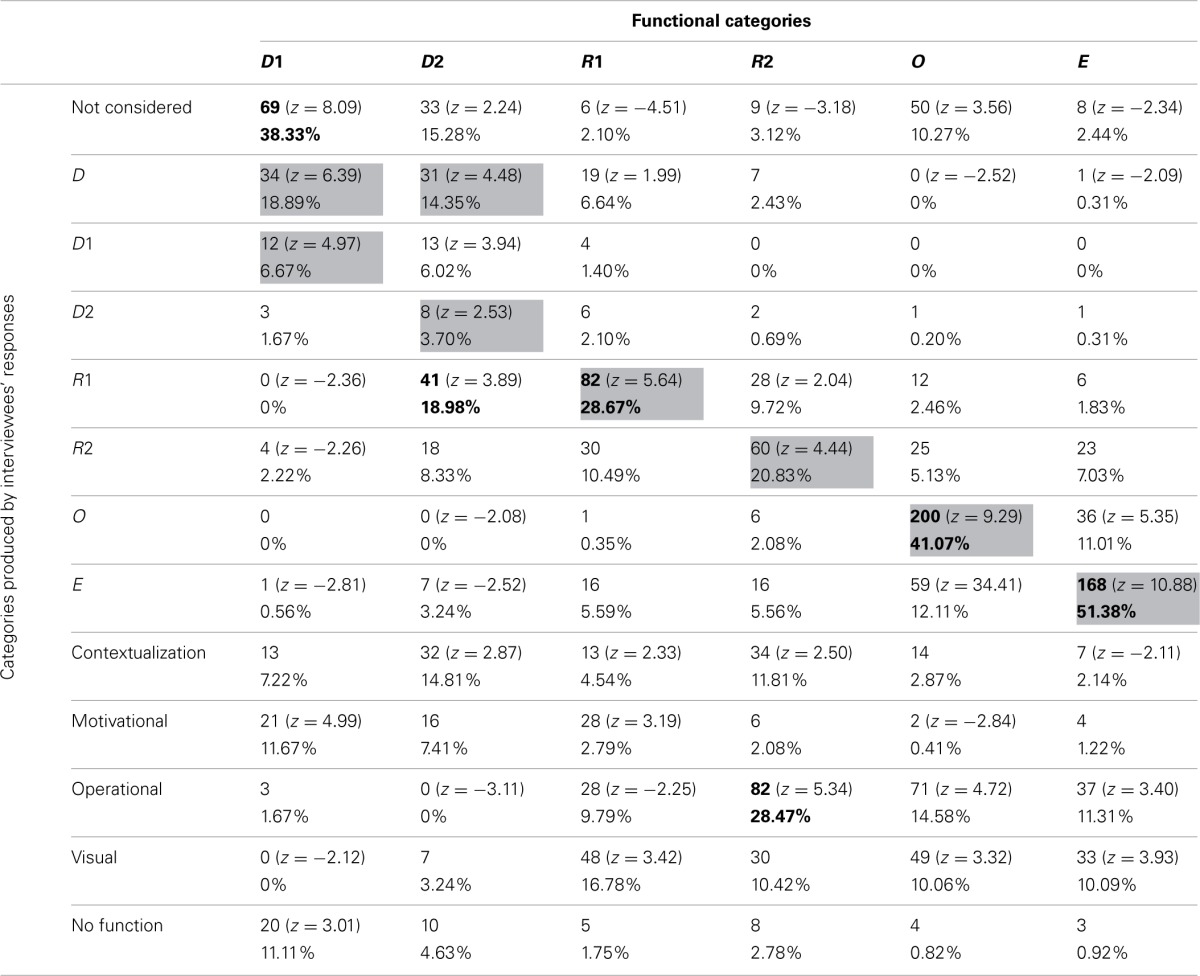
**Frequencies and percentages of category attribution for students**.

**Table 10:**
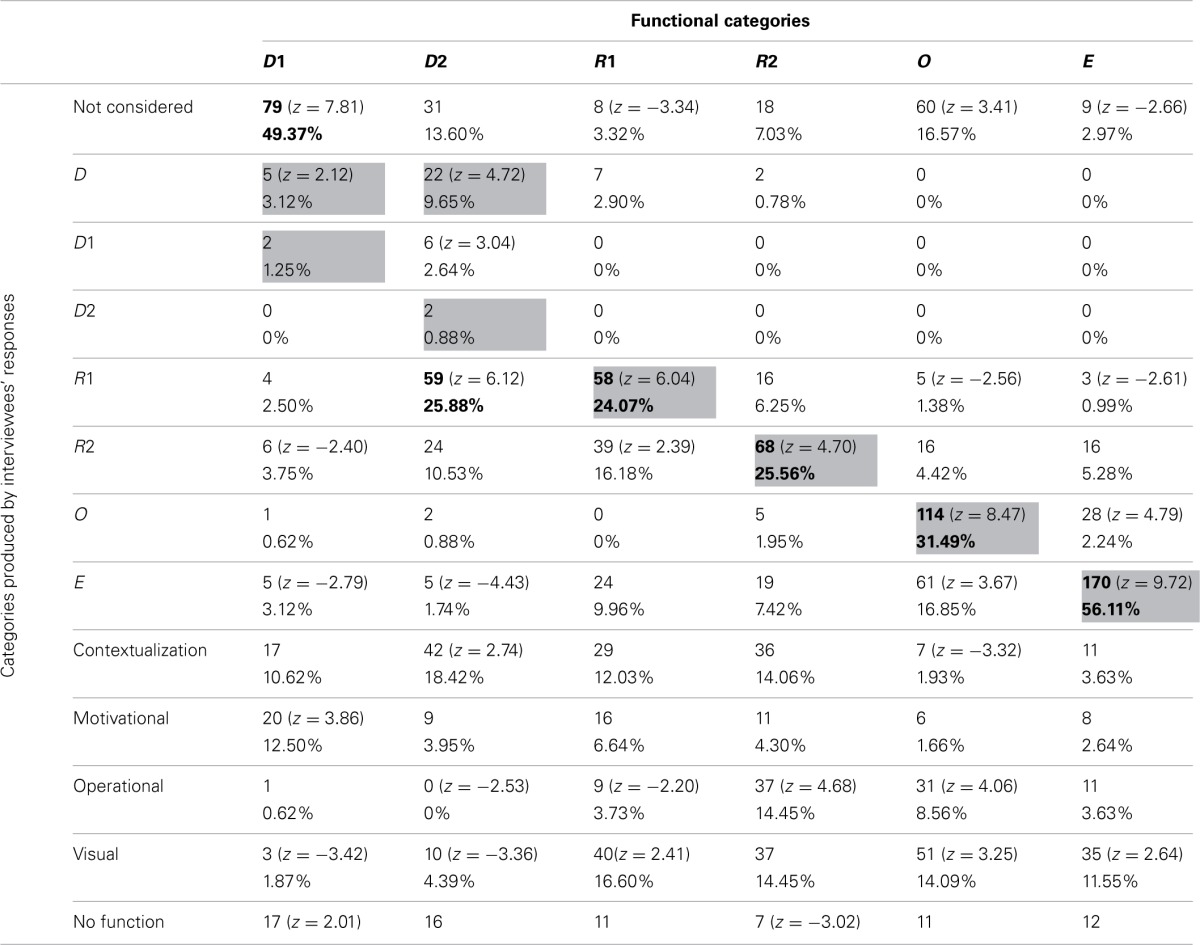
**Frequencies and percentages of category attribution for the control group**.

The groups did not differ greatly from the general tendency of the whole sample. Nevertheless some differences, which explain the results of the log-linear analyses, can be noted. All the four groups generally tended not to consider D1 pictures. However, whereas teachers, illustrators, and control participants had a high score in the M category, students were the only group who appeared to prefer the Decorative category (18.89% of students described as Decorative pictures that were not even considered by 38.33% of other participants). This was consistent with Mayer's categorization, since our sub-samples tended not to consider those pictures that, according to Mayer, have no cognitive function.

Also D2 pictures were considered differently by the four groups. Teachers tended not to consider them, equating them with the D1 pictures. Illustrators saw them mainly as C pictures (19.27%). It is worthwhile noting that ascribing Decorative pictures to this category was a common tendency of the whole sample, even though the other subsamples did not use this category as often as the illustrators did. Students and control group participants tended to mistake Decorative pictures for R1 images.

R1 pictures tended to be correctly detected by all groups. The teacher group was once again the only one that regularly hypothesized an emotional-motivation function. For the other groups, the MN category appeared to be one of the most used.

R2 images tended to be correctly detected by all groups. Since many pictures in this category were used to illustrate exercises, the OP category was also often used by participants. Illustrators, unlike other participants, tended to mistake R2 images for E pictures (13.33%), showing that these individuals sometime overrate the cognitive function of such images. The control group showed a tendency to positively evaluate the visual impact of those pictures that often stand in for the text—and this is proved by their rating R2 images as C (14.09%) or MN pictures (14.45%).

O pictures were correctly categorized by all groups except for the illustrators, who often mistook them for E ones. Thus, the tendency of this subsample to overrate the cognitive function of the pictures was confirmed once more. It is also interesting to note that all the participants, apart from the students, tended (when not assigning them to the right category) not to consider the O pictures as proper illustrations. This can be explained by the fact that many O pictures were schemata or graphs, and thus possibly more familiar to students than to other groups. Teachers and students, who are more used to employing the kind of illustrations analysed in the present study for their practical work than the other participant categories, also used the OP category to describe those pictures.

E pictures seemed to be the most easily and univocally recognized, gaining more than 50% identification in every group.

Even if strong differences among subsamples failed to emerge, it is possible, nonetheless, to locate some tendencies. In general, whereas illustrators and teachers tended to have differing opinions, students and the control group often seemed to converge in their evaluations/interpretations. These last two subsamples tended to agree with Mayer's theories more frequently than did the others. Moreover, illustrators tended to overrate the cognitive function of all pictures, whereas teachers tended to stress the Motivational functions of the Decorative and Representational pictures.

#### Pictures' utility

A 6 × 4 MANOVA was conducted to explore the differences among the four subsamples in the attribution of utility scores (Table 11). The mean score attributed to D2 pictures was higher for illustrators and this difference was significant: *F*_(3, 64)_ = 2.89, *p* < 0.05. The mean score attributed to Organizational illustrations was higher for students and this difference was also significant: *F*_(3, 64)_ = 3.27, *p* < 0.05. *A posteriori* Turkey tests showed that the mean scores attributed to O pictures differed significantly between illustrators and students (*p* < 0.05). No other difference was statistically significant.

**Table 11:** **Mean scores and *SD*s in the different subsamples**.

**Category**	**Subsample**	**Mean**	***SD***
Decorative 1	Teachers	1.94	0.17
	Illustrators	2.21	0.18
	Students	1.69	0.17
	Control	1.75	0.18
Decorative 2	Teachers	2.87	0.13
	Illustrators	3.34	0.13
	Students	2.87	0.13
	Control	3.05	0.13
Representational 1	Teachers	3.98	0.11
	Illustrators	3.95	0.12
	Students	3.95	0.11
	Control	3.96	0.12
Representational 2	Teachers	4.01	0.11
	Illustrators	3.85	0.12
	Students	4.13	0.11
	Control	3.94	0.12
Organizational	Teachers	3.76	0.13
	Illustrators	3.40	0.14
	Students	3.92	0.13
	Control	3.51	0.14
Explanative	Teachers	4.21	0.12
	Illustrators	4.12	0.13
	Students	4.42	0.12
	Control	4.28	0.13

## Discussion and conclusions

Up to now we have no empirical data about the conceptions that people possess about text-picture combinations devised for educational purposes. The present study was carried out to fill such a lack of data by trying to thoroughly investigate people's beliefs regarding the role and effectiveness of illustrations in children's textbooks. Our aim was to record participants' opinions by asking them to consider concrete cases. An open interview, based on specific pictures taken from actual textbooks, secured more trustworthy data than that obtained with less ecological methodologies, also due to a greater ecological validity of the employed materials. Considering the fact that beliefs are often not available as abstract conceptualizations, we did not ask participants to provide only verbal answers, but also requested a response through practical actions, such as selecting pictures and positioning them to create an original product.

Three main issues were addressed in the study: (1) the description of the conception about multimedia learning which emerged from participants' responses, (2) the internal consistency of such conception, and (3) the possible differences in such conception depending on the level of the respondents' expertise.

### Features of the conception about multimedia learning

As far as the first issue is concerned, participants' responses allowed us to extend the frame in which beliefs about multimedia learning can be conceptualized. More precisely, the study led us to identify new categories, to use alongside those devised by previous authors, in which pictures can be classified according to their functional role described in terms of the mental processes which the text-picture combination activates. As a result, a comprehensive and systematic set of kinds of illustrations, each associated with the corresponding cognitive mechanism, is proposed.

Two categories proved to be the most interesting: on the one hand, the Motivational category, and, on the other hand, the Mnemonic. The first one converges with a cultural-pedagogical position (Korat, 2001), which ascribes importance to a strong link between social-emotive aspects (self-identity, use of narratives linked to a child's life, social interaction) and cognitive skills to promote a child's literacy. This correspondence might mean that “non-expert” people have deep-rooted naïve theories about themes related to cognitive psychology, and that these theories—whereas diverging from those accredited by cognitive psychology—have a rationale of their own. The Mnemonic category, on the other hand, has an iconic function, known as the transformation function, which was identified by Levin (1983), but later disregarded by Mayer (1993). According to Levin, pictures related to this category are designed to facilitate the memorization of the text to which they refer. The visual recoding of the information allows the reader to spontaneously use some sort of visual mnemonic technique. Pictures focus the attention on the main points of the content to be acquired, thus providing people with methodical ways to memorize data and making the data more concrete and storable. Levin (1983) also claimed that such pictures link different and distinct parts of information, forming a well-organized global context. This last description seems to be suitable for those Organizational pictures (such as geographical maps or hierarchical structures), which many interviewees put into the Mnemonic category. The same definitions employed by the interviewees to denote this category recall the key words used by Levin (1983). For example: «The picture promotes the visualization», or «This picture allows the learner to recall the corresponding text», or even «The illustration gives a prompt idea of the content and helps to memorize it».

The overall view which emerged allowed us to draw a complete taxonomy of the functions that pictures play in multimedia instructional tools supported by empirical evidence. Such a taxonomy can be described by highlighting the cognitive processes which underpins such functions. The taxonomy can be conceived as hierarchically organized, moving from the analysis of what illustrations allow learners to represent in their mind (something which is mentioned versus not-mentioned in the text) to the features of such a representation (restricted to the visualization of a single element versus including also relationships with other elements) and, finally, to the goal of such representation (just displaying the whole system of elements mentioned in the text versus explaining the dynamics of such a system).

### Internal coherence of the conception about multimedia learning

Participants' responses to different requests concerning the functions and utilities of pictures were always coherent, emphasizing a systematic nature of their beliefs. In particular, a strong coherence between the perceived utility among the three requests emerged. The equal use of Representational and Motivational pictures in building the Perfect Book mirrored the positive judgment people ascribed to them and the ease with which the participants identified the pictures' role in education.

Thus, we can argue that the beliefs concerning multimedia learning are well-structured—so as to constitute a sort of naïve theory in which opinions about the mental processes elicited by text-picture combinations are consistently associated with opinions regarding the cognitive functions they play and their efficacy within the general economy of mental work implied in learning.

This coherent view developed by non-expert persons is not so far from Mayer's theory. According to this author, laypersons develop beliefs about the mechanisms of multimedia learning (the so-called common sense theory), which conflicts with what is supported by experimental research. According to the common sense theory of multimedia learning, pictures are included in instructional materials just to convey information and they have no added value. In contrast to this view, our data supports the notion that people (i) identify further, and deeper, functions played by illustrations, (ii) associate these functions with the specific underlying cognitive processes (iii) which are meant to be addressed to specific goals (expressed in psychological terms: amusing, memorizing, understanding, and so on), (iv) so as to be able to judge properly how useful pictures can be in order to enhance or impede learning.

### Differences among subsamples concerning the conception about multimedia learning

Strong differences between the interpretations of multimedia experts and non-experts failed to emerge, thus supporting the notion that folk ideas concerning multimedia learning are quite generally shared and robust. It was possible, nonetheless, to highlight some significant differences. In general, while illustrators and teachers tended to have differing opinions, students and the control group often seemed to converge.

These last two subsamples tended - more frequently than the others - to agree with Mayer's theory. This data seems to be particularly relevant. Mayer, in fact, predominantly employed university students as his subjects. From our study the idea that people have naïve theories that correspond to the common sense theory (as Mayer—2001, 2005—hypothesized) seems to be refuted. On the contrary, it appears as if this sub-sample comes very close to the core concept of the multimedia theory, in which the role of individual beliefs is important, even more since they are quite adequate with respect to multimedia principles. Moreover, the study helps to understand how much of Mayer's multimedia theory is spontaneously recognized by non-experts and by people employed in the didactic field.

### Naïve conceptions about multimedia learning and Mayer's common sense theory

The naïve conceptions of psychological mechanisms involved in multimedia learning, which were investigated in the present study, allow us to point to a fairly high correspondence between people's views and Mayer's model. This is especially true for those categories (O and E) that seem to have the greatest influence on cognitive processes. Decorative and Representational pictures, on the other hand, are more often ascribed to different and naïve categories. Concerning the attribution of function to different images, data leads us to infer that the pictures that, according to Mayer, involve more complex cognitive processes (such as O and E pictures), were recognized with greater ease by the participants. However, the opposite was true for the pictures that, in Mayer's classification, do not have any cognitive function: subjects tended to ascribe different and “personal” functions to these decorative pictures. By examining the participants' utility scores, one can see that D1 pictures were considered to be less useful, while the E pictures appeared to be perceived as being the most useful. The most and least useful pictures endorsed by interviewees were always coherent with Mayer's functional classification: E pictures were regarded as the most useful, and Decorative as the least useful. Examination of the Perfect Books built by participants revealed sound consistency with Mayer's principle of special contiguity: this correspondence was maintained in 100% of the cases.

The match between people's conception and Mayer's experimentally supported theory may be attributed to several factors, not least because Mayer often focused his research on scientific disciplinary fields or on differences between the American and Italian educational systems and cultures. Moreover, historical factors cannot be disregarded; the studies, allegedly supporting Mayer's pessimistic view about the common sense theory (Mayer, 2001), were performed more a decade apart, and a more widespread diffusion of multimedia tools in everyday life could have provided people with the opportunity to gain a broader and more sophisticated life experience, and, consequently, a better understanding of multimedia principles.

### Implications

On a practical level, this study appears to be useful for several reasons. First of all, it is important to consider the naïve theories of graphic artists who are active participants in the realization of textbook illustration. If their conceptions are “wrong” (that is, not coherent with cognitive theories, and hence referring to misleading cognitive principles), the illustrators themselves will contribute to the creation of cognitively dysfunctional textbooks.

Teachers' conceptions are equally important, as it is teachers who actually choose the textbooks and decide how to use them in classroom. Thus, an adequate conception is important to guaranteeing a good use of pictures as a cognitive aid (e.g., being able to point out to students which pictures it can be useful to focus on, which pictures are to be studied, which are to be explained in relation to the text, and so on).

Finally, students, if they possess relevant systems of beliefs, can be conceived as competent self-regulated learners, who are able to decide by themselves which illustrations they must pay attention to according to the goals they should reach and to identify the mental work which is associated with each kind of image. Furthermore, the possession of an adequate conception should enable learners to devise efficient multimedia presentations, a task which is increasingly assigned to students in contemporary instructional settings.

This study provided suggestions to involve instructional tool designers, teachers and students in reflections about the adequacy of the multimedia tools which they manage. Firstly, a tested framework to distinguish among different kinds of illustrations is provided. Secondly, the procedure (as well as the kind of tasks and questions) used in this investigation can be applied in school settings to highlight the implicit ideas teachers and learners have about the alleged effects of multimedia devices and to verify if they match the cognitive principles supported by empirical research. Thirdly, the naïve conception which emerged in this study can be assumed as a reference point to question the conception that teachers and students share and to hint at discussing it.

Another interesting implication can be derived by the additional categories added to the original taxonomy, and derived from participants' answers. Many of these categories, as discussed above, are closely related to everyday learning practice, and clearly derived in a cognitively sensible way from participants' expertise. Insights from these new categories should be operationalized both by multimedia designers and users' of multimedia products. For example, the first “new” categories—contextualizing and motivational pictures—takes into account the socio-emotional side of the learning process, disregarded by traditional cognitive theories. In everyday learning, though, this attention could be extremely useful to promote effective learning. The other two categories named by our sample, operational and mnemonic, are more on the cognitive side, but should be used as useful hints by designers, because they stress two important steps of the learning process, especially in the early literacy steps: operationalize complex procedures/concepts and memorize information. Being able to direct effectively the use of multimedia elements to would be extremely useful.

### Limits and further steps

We have to take into account the limitations of this study. It was a preliminary attempt to analyse naïve conception associated with the use of multimedia tools for instructional aims. Since no previous investigation focused on beliefs about the cognitive aspects of text-picture presentation has been carried out, we had no specific theoretical or methodological model to be taken as a frame of reference. We followed a general phaenomenographic approach (Marton, 1981) aimed at inducing respondents to express the sense they attribute to that piece of experience which concerns multimedia learning and the deep convictions they possess about it. On the methodological side, we devised an original procedure to address this issue. We tried to combine different procedures, ranging from verbal to concrete tasks, as well as from open to closed questions (in the form of both dichotomous selections and Likert scales). We hoped to facilitate the interviewees in expressing their conceptions by means of various, complementary ways of answering, so compensating for the limits of each procedure. On the other hand, however, we made a precise methodological choice in deciding to reconstruct the participants' view by making reference to specific instances of text-picture presentation, but not to ask questions about multimedia tools in general, since we thought that the former, but not the latter procedure would enable us to go beyond superficial opinions and to test deeply the respondents' convictions. Obviously this makes the investigation rather contextualized, so raising problems with the generalization of the findings. The fact that the most widely adopted textbooks were selected and that a large set of text-picture combinations was employed in the study should assure that the material considered here was representative of the instructional tools usually employed in school settings, so mitigating the limitation in question. However, the issue of contextualization can be meant also in another sense. Metacognitive knowledge in itself is contextualized (Annevirta and Vauras, 2006; Anderson et al., 2009) in the sense that beliefs about the optimal way to combine texts and pictures may vary according to the specific learners' goals, skills, and habits, as well as according to teachers' and tasks' requests.

This study provided a first overview of the naïve conceptions about text-picture combinations. Further studies might investigate this topic by testing whether these conceptions are influenced by the type of experience people have of multimedia tools and by the levels of previous knowledge and ability, as well as by cognitive styles. Another interesting evolution of the present study, from a methodological point of view, could be to vary the goals given to the participants while rating the pictures, asking individuals randomly to rate them imagining a use linked “to study” vs. “to design good text” and see whether differences emerge between the designers and the learners. An interesting way to deepen this issue even further in future research could be by exploring not only beliefs, but also how beliefs are related to use of learning materials, using a mixed method design. Another natural development of the present study would be of applying a similar methodology to multimedia instructional material presented using different kind of devices: would individuals' conception change if they are requested to evaluate an illustration presented as printed illustration in a textbook, or if the same picture is presented within a multimedia didactic tool, a blog, a social media or within an instructional application for a tablet?

### Conflict of interest statement

The authors declare that the research was conducted in the absence of any commercial or financial relationships that could be construed as a potential conflict of interest.

## Footnotes

^1^This classification was compiled by Italian Ministry of Education on the basis of the choices made by teachers and communicated by schools to the Ministry itself, which thus constitutes a valid source of this kind of data.

^2^To better explain our methodology in this first phase of the research, it is important to note that in Italian primary schools there is a difference between the structure of the first two grades (first cycle, where a more integrated structure of disciplines is planned) and the last three grades (second cycle, where, as a kind of transition to a more advanced grade, a distinction between disciplinary subjects and reading activity is made). This distinction is reflected in the textbooks: whereas texts for the first two grades are often conceived as a whole (even though they are sometimes made up of several volumes, for teachers' and pupils' convenience of use), texts for the last three grades are divided into two different units: disciplinary subjects and reading activities. The distinction is quite clear-cut, especially for the last two years when, to suit the increasing learning demands of the growing pupils, the different units are grouped into different textbooks, so that often books from different publishing houses are chosen for the two areas in the same class. For these classes, textbooks both for disciplinary subjects and for reading activities were examined in the survey.

^3^A page with one illustration only was also obviously included. This was done to balance the spontaneous comparison among pictures in the same page with the evaluation of single illustrations.

^4^Originally it had been proposed to use a five-point scale from 1–5. After an exploratory preliminary administration, participants were asked, at the end of the interview, for comments on the procedure. It was noticed that the interviewees who had difficulty rating the pictures were seemingly unable to calibrate their judgements. To facilitate this task the researchers decided to transpose the scale using values from −2 to +2, hypothesising that the difference between positive and negative values could help the respondent. Another pilot phase confirmed this hypothesis. Therefore this version of the scale was maintained in the main study.

^5^Two other categories (0 and 12) were coded. They indicate, respectively, a picture that was not considered and an explicit statement of the considered illustration's lack of function.
